# Safety and efficacy of a pre-wrkout dietary supplement with and without synephrine

**DOI:** 10.1186/1550-2783-12-S1-P5

**Published:** 2015-09-21

**Authors:** R Dalton, YP Jung, C Rasmussen, P Murano, CP Earnest, RB Kreider

**Affiliations:** 1 Exercise & Sport Nutrition Lab, Texas A&M University, College Station, TX, USA; 2 Institute for Obesity Research & Program Evaluation, Texas A&M University, College Station, TX, USA; 3 Nutrabolt International Inc., Bryan, TX, USA

## Background

A number of nutritional strategies have been developed to optimize nutrient delivery prior to exercise. As a result, a number of pre-workout supplements have been developed to increase energy availability, promote vasodilation, and/or positively affect exercise capacity. The purpose of this study was to examine the safety and efficacy of a pre-workout dietary supplement with and without synephrine.

## Methods

In a double-blind, crossover, randomized and placebo-controlled manner; 25 apparently healthy and recreationally active men and women (21.76 ± 3.00 yr, 15.24 ± 5.26% fat, 25.09 ± 3.03kg/m^2^) had the first blood donation after 10-12 hours fasting, and then after 2 hours of a pre-workout supplement ingestion, participants had the second blood donation. Participants ingested in a randomized and counterbalanced manner a dextrose flavored placebo (P); a pre-workout supplement (PWS) containing 3g beta alanine, 2g creatine nitrate, 2g arginine AKG, 300mg N-acetyl tyrosine, 270mg caffeine, 15mg *Mucuna pruriens*; or, the PWS with 20mg synephrine (PWS+S). Participants repeated the experiment after a one week washout period with the alternate supplements in a randomized and counterbalanced manner. Data were analyzed by repeated measure ANOVA and presented as means (95% CI) delta change from baseline.

## Results

Delta analysis revealed significant differences among groups in mean change in blood urea nitrogen (BUN) (unit conversion to mg/dl by mmol/l × 2.8011): P (-1.51mg/dl; -2.26, -0.78), PWS (-2.26mg/dl; -2.99, -1.54), and PWS+S (-0.56mg/dl; -1.28, 0.14), creatinine (CRE) (unit conversion to mg/dl by µmol/L × 0.0113): P (0.05mg/dl; 0.01, 0.10), PWS (0.14mg/dl; 0.09, 0.19), and PWS+S (0.14mg/dl; 0.09, 0.18). An overall Wilks' Lambda time (p < 0.01) and time × group (p < 0.01) interactions for BUN, CRE and the ratio of BUN/CRE (BCr), and Greenhouse-Geisser univariate analysis for BUN, CRE and BCr (p < 0.01) were found. Wilks' Lambda analysis revealed a significant time effect (p < 0.05) of alkaline phosphatase (ALP), aspartate amino transferase (ALT), and alanine amino transferase (AST), and of creatine kinase (CK) and lactate dehydrogenase (LDH), with no time × group interactions (p > 0.05). MANOVA Greenhouse-Geisser univariate analysis revealed significant changes over time for ALP, ALT and AST (p < 0.01), and CK and LDH (p < 0.01). Delta analysis revealed significant differences among groups in mean change in total cholesterol (CHOL): P (0.31mmol/L; 0.12, 0.50), PWS (-0.16mmol/L; -0.35, 0.02), and PWS+S (0.31mmol/L; 0.12, 0.50). An overall Wilks' Lambda time (p < 0.01) and time × group (p < 0.01) interactions for CHO, HDL-C, LDL-C and triglyceride (TAG), and Greenhouse'Geisser univariate analysis for CHO, HDL-C, and LDL-C (p < 0.01) were found. Delta analysis revealed significant differences among groups in mean change in glucose: P (0.60mmol/L; 0.21, 0.99), PWS (0.77mmol/L; 0.39, 1.15), and PWS+S (1.29mmol/L; 0.90, 1.68). A significant time × group interactions (p < 0.03) of glucose was found.

## Conclusion

Ingesting a dietary PWS or PWS+S had minor affects within 3 hours, similar to P, on kidney function, liver enzymes, blood lipid levels, muscle enzymes, and blood sugar levels. These findings are in agreement with other studies testing similar ingredients.

